# The gap between the need for parental support and support provided during the COVID-19 pandemic: a qualitative study with first-time mothers’ experiences

**DOI:** 10.1186/s12889-024-20520-x

**Published:** 2024-10-29

**Authors:** Heléne Appelgren Engström, Marie Golsäter, Maria Harder

**Affiliations:** 1 School of Health, Care and Social Welfare, Mälardalen University, Västerås, Sweden; 2 ChiP Research Group, School of Health, Care and Social Welfare, Mälardalen University, Västerås, Sweden; 3 CHILD Research Group, School of Health and Welfare, Jönköping University, Jönköping, Sweden; 4 The Child Health Care Service and Futurum – Academy for Health and Care, Region Jönköping County, Jönköping, Sweden

**Keywords:** Child Health Services, COVID-19 pandemic, first-time mothers, parental support, qualitative

## Abstract

**Background:**

Becoming a parent can be challenging. Becoming a parent for the first time during the COVID-19 pandemic might pose additional challenges, as the pandemic has imposed restrictions on society, thus affecting parental support. There were changes in parental support from child health services and preschools available for all children and their parents, so called open preschools, have been closed. This study explored first-time parents’ experiences of the parental support they received during the COVID-19 pandemic.

**Methods:**

A qualitative study involving individual semi-structured interviews with nine first-time mothers who had been on parental leave during the pandemic was conducted. Data were analyzed with inductive content analysis and the results are presented in a main category with three generic categories.

**Results:**

The main category is entitled, *A gap between the needs of parental support and the support provided*, and it encompasses three generic categories: The first category, *Formal support*, refers to support from child health nurses and open preschools. The second category, *Lack of formal support*, encompasses the lack of person-centred support and lack of parental groups. The third category, *Informal support*, encompasses support from family, friends, and social media.

**Conclusions:**

The findings indicate that under the restrictions imposed by the pandemic, first-time mothers expressed the need for person-centred support to both parents which will ensure that all parents get the support they expect and need. The participants also expressed a desire for adapted parental groups that are feasible despite the restrictions to allow them to connect with other parents and build networks.

## Background

Becoming a parent and having a child can be both highly anticipated but also challenging. Becoming a parent for the first time during the COVID-19 pandemic might be different from what is expected and pose additional challenges [1–3]. The pandemic has imposed restrictions on society with social distancing and changes in parental support. Therefore, first-time parents have had to manage the transition to parenthood with less support [2–5]. Consequently, it is important to understand first-time parents’ experiences of parental support during the COVID-19 pandemic.

In Sweden the Child health services (CHS) offer parental support as a part of the national child health care program [6]. The aim of this programme is to monitor children’s health, development, and living conditions and offer parental support individually and in groups. Further, it aims to provide accessible, equal and fair child health care for all children and their parents. The service is free of charge. The program is conducted within children’s age range of 0 to 6 years [7] and reaches approximately 99% of the children and their parents [8]. To achieve the goal, the child health service offers universal interventions to all children and their parents as well as targeted interventions when such needs emerge [6] as when parents need extended support due to vulnerability [9]. On an individual level CH nurses offer universal health counselling and guidance to support and empower parents in their parenting in their everyday life. Further, all mothers are screened for depression and those who expresses worries, or anxiety are provided with support as a targeted intervention [6]. Another universal intervention is parental support in groups within antenatal and CHS settings to support expectant and new parents and provide prerequisites [10] for them to develop their social network and knowledge about the child’s rights, health, and development to strengthen them in their parenting [11]. Early parental support and early interventions for parents can promote health and well-being as well as prevent ill health in both children and parents [11]. Another arena for parental support is the preschools available for all children (0–5 years of age) and their parents, so called open preschool, which is a meeting place free of charge [6]. In general, there is a close connection between the CHS, and the open preschools and they can also be located in the same building, a family centre [9].

A recent published review [12] state valuable insights on how to facilitate the transition to parenthood; early interventions to promote mental health, improve the parent-infant relationship, and enhancing parenting skills. A study from the Netherlands concluded that parents with a child aged 0–2 years of age asked for a more person-centred support from healthcare professionals although they appreciated the support they received [13]. Another study [14] reported that evaluative support and informational support is significantly related to confidence in infant care. Public health professionals e.g. CH nurses are the professionals reported by most first-time mothers to have provided informational and assessment support [14]. Research exploring first-time parents’ transition to parenthood shows that although first-time mothers felt they had prepared for motherhood, both on their own and by attending childbirth classes, the reality of motherhood was different than expected [15]. Furthermore, first-time parents felt that their relationship was influenced by their child as only the child was in focus [16]. Although they appreciated professional support, they saw this support as for the child. They found little support for dealing with mental health and relationship problems [17].

The COVID-19 pandemic has resulted in changes in society, including placing restrictions on physical and social distancing, and led to adjustments in parental support from the CHS and the open preschools [5, 18]. These adjustments meant that open preschools were closed and the number of visitors within CHS was limited to only one parent per visit [1] usually both parents are invited to health visits. Furthermore, parental groups during pregnancy and after childbirth were cancelled [1, 19, 20] and replaced with individual visits [1].

The advice on physical and social distancing has contributed to a decrease in informal social support, and parents have become increasingly socially isolated during the pandemic [1, 5, 18]. This study provides some knowledge and understanding of how first-time parents have experienced parental support during the COVID-19 pandemic. This study will thus allow further development of existing parental support systems as well as knowledge-enhancement about the continued support needs of parents who had their first child during the pandemic.

### Aim

This study aimed to explore first-time parents’ experiences of parental support during the COVID-19 pandemic.

## Methods

A qualitative study based on telephone interviews with first-time parents was conducted to explore their experiences of parental support during the COVID-19 pandemic.

### Recruitment of participants and data collection

This study was conducted in two regions of Sweden between February and April 2022. The inclusion criterion was first-time parents who had been on parental leave during the COVID-19 pandemic. Parents were recruited by asking for contact details in a questionnaire about parenting handed to them by CH nurses. In total, twelve parents left their contact details, and further contact was established with nine of them, all mothers. The dropout of three parents were due to them not calling back after two left messages on the answering machine and one parent who did not show up for an interview appointment and then did not call back after a message on the answering machine. The nine mother participating in the study had been on parental leave during the pandemic. These nine mothers ranged in age from 25 to 35 years. Eight participants had one child, and one participant had two children during the pandemic. The children’s ages were between five months and two years. Seven participants had a university degree and two had a high school diploma. All participants lived together with the other parent.

A semi-structured interview guide was developed to explore first-time parents’ experiences of parental support and what support needs they had during and after the COVID-19 pandemic and how these needs were met. Due to the pandemic, the interviews were held over the telephone by the last author who has experiences from the CH services and extensive experience in conducting qualitative studies and interviews. The interviews lasted between 32 and 72 min. These interviews were recorded and then transcribed verbatim.

### Data analysis

There are a limited number of studies about mothers’ experiences of parental support during the COVID-19 pandemic. Therefore, a qualitative inductive content analysis was deemed suitable. Content analysis [21] comprises three phases: preparation, organisation, and reporting. The first step was to get familiar with the data by reading and rereading the transcribed interviews. In the second step, simplified codes were identified, by writing notes in the margins to organise the data. The codes were compared through a continual discussion between the first and the last author. These codes were first grouped into sub-categories and then grouped into generic categories describing similarities and differences. See Table 1 for an example of the analysis process. The abstraction process continued until the main category, *A gap between the needs of parental support and the support provided*, was identified. The second author read and commented on the analysis and final findings. The last step was to report the findings through three generic categories with clarifying quotes.

**Table 1:** An example of the analysis process

Code	Sub-category	Generic category
Neglecting parents’ mental and/or physical needs		
Lack of support to both parents	Lack of person-centred support	
		Lack of formal support
Lack of information	Lack of parental groups	
Lack of social interaction		

### Ethical considerations

This study was approved by an ethical committee (Dnr 2021–04843) and designed, conducted, and reported in accordance with national requirements [22] and the principles of the Declaration of Helsinki [23]. Participants were informed about the study. They participated voluntarily and could end their participation at any time. Where citations are given in the results, participants’ names are not revealed.

## Results

The first-time mothers’ experiences of parental support during the COVID-19 pandemic can be described as *a gap between the needs for parental support and the support provided.* These mothers described their need for person-centred support which does not always correspond to the formal support provided. Individualization of the support seemed to be significant during the pandemic-related societal restrictions as the first-time mothers lack other social networks usually available for first-time parents such as parental groups, family, and friends. Furthermore, the first-time mothers described a lack of support for themselves and their partners. The focus was mainly on the child’s health and development. The main category, *A gap between the needs for parental support and the support provided*, includes three generic categories: *Formal support*, *Lack of formal support*, and *Informal support* (see Table 2).

**Table 2:** Overview of the results

Sub-category	Generic category	Main category
Support from CH nurses Support from open preschool	Formal support	
Lack of person-centred support Lack of parental groups	Lack of formal support	A gap between the needs for parental support and the support provided
Support from family and friends Support from social media	Informal support	

### Formal support

First-time mothers’ experiences described as formal support refer to the support society offers. In a non-pandemic context, parents may receive parental support from CH nurses as well as from parental groups and open preschools. During the COVID-19 pandemic, the first-time mothers’ experiences of formal support are described through the sub-categories *Support from CH nurses* and *Support from open preschool*.

#### Support from CH nurses

The first-time mothers’ experiences of support from CH nurses contain descriptions of being safe and receiving parental support. Being safe refers to the notion that CH nurses provided safety and acted in a welcoming manner and made exceptions to societal restrictions imposed due to the pandemic by allowing both parents to attend health visits.

*‘Then said …. our CH nurse*,* to us or to me that we were both welcome… I thought she did that to everyone*,* but after two*,* three months we understood that there she also made an exception to what these regulations really are… The father is not allowed to join’* (i5).

Receiving parental support refers both to the support first-time mothers received from CH nurses during the health visits at the CHS and the support received from CH nurses and counsellors during home visits. The mothers said that it was important for them that the CH nurses were available to answer their queries during the pandemic. *One mother expressed: ‘We have a good nurse now and we get good advice when there is something with the child. I feel she listens to us’* (i9). The support received is described as having information and being given advice concerning food, everyday routines, and how to handle teething and colds. Further, receiving parental support encompasses having regular follow-ups with both parents and/or the whole family’s wellbeing during the COVID-19 pandemic. However, there were first-time mothers who related experiences where the nurses presupposed that all parents had been socially isolated during the pandemic even when the mothers indicated otherwise.

#### Support from open preschools

The first-time mothers’ experiences of support from open preschools relate to activities during the pandemic and the opportunity of getting out and breaking social isolation after the lockdown. Activities during the pandemic refer to the first-time mothers’ descriptions of activities organized by employees in open preschools. The parents described how the activities were adapted to the pandemic situation. For example, instead of meeting indoors at the preschool, pram walks to create social connections were arranged. One mother said:

*‘No*,* they did what they could so that - when it was spring there*,* they had pram walks. I was also involved in that. Then we walked around a lake and talked. A preschool teacher was with us and a counsellor was with us and we split into groups so that there weren’t too many of us and then we walked around the lake and talked. It was also such a social thing’* (i6).

The first-time mothers also described their appreciation of other activities that were offered, such as digital music and singing sessions. However, there were first-time mothers who said that they did not need the support available at an open preschool.

Breaking social interaction after the lockdown refers to descriptions of how an open preschool provides opportunities for socializing with other parents and children. The first-time mothers made new contacts with other parents through preschool. However, other first-time mothers said that preschool became a meeting place for the moment but did not contribute to new relationships. Further, they described the preschool teachers as being a source of support, available to answer their everyday questions.

*‘So*,* in that way*,* it’s a bit of a vent to still meet a little*,* both other mothers and these preschool teachers who came’* (i1).

### Lack of formal support

The lack of formal support pertains to the experiences of some first-time mothers who felt that some elements of formal support was absent during the COVID-19 pandemic. This is described through the sub-categories *Lack of person-centred support* and *Lack of parental groups*.

#### Lack of person-centred support

The first-time parents’ experiences of lack of person-centred support refer to the notion that the support from CHS was too general and too focused on the child, neglecting the parents’ mental and/or physical needs and lack of support to both parents. Regarding neglecting mental needs, first-time mothers described instances where their desire for mental support was not acknowledged. Being a first-time parent necessitates making adjustments to everyday life, and support for one’s mental needs is important for the family in the long term. Regarding neglecting the parents’ physical needs, the first-time mothers said that the CH nurses only focused on the child’s development and that the first-time mothers did not receive help with their physical problems after childbirth. The mothers felt forgotten when the baby was born. For example, one mother wished that a CH nurse had asked how the parents were doing instead of only weighing and measuring the child; *‘*…*above all*,* I think that the mental check-in with both the mother and the partner can be at least as important as weighing the child every two weeks sometimes* (i7)*’*.

When their needs are not met, first-time mothers describe feeling unseen as individuals. One mother expressed: *‘They haven’t been unsupportive*,* but… to general advice*,* I think it’s not… so individualized*’[i2]. Lack of support to both parents refer to first-time mothers expressed feeling completely alone and without support when their partners were not allowed to attend appointments during the pandemic.

#### Lack of parental groups

The first-time mothers’ experiences of lack of parental groups revolve around not being invited to physical parental groups. Even when the physical groups were replaced with digital groups, the parents experienced lacking information and lacking social interaction. Lacking information refers to the first-time mothers’ descriptions of lacking an opportunity to get parental education, for instance about childbirth and breastfeeding. The first-time mothers stated that replacing content from parental groups with information films was not enough as they then did not have an opportunity to ask follow-up questions. Further, the first-time mothers said that the flow of information (during pregnancy) during the pandemic was inconsistent and unstable between ward units, for example, regarding vaccinations and lectures before childbirth. This inconsistency and lack of stability caused insecurity that persisted during labour and parenthood. The first-time mothers also reported a lack of information directed at the whole family, which necessitated their own information search during the pandemic.

Lacking social interaction refers to the fact that first-time mothers lacked opportunities to meet and create relationships with other parents in similar situations during the pandemic, something they had anticipated during pregnancy. One mother reflected: ‘*it’s a bit sad*,* because my husband didn’t get to be so involved… he was not allowed to come with me to the maternal health care… …had thought that we would go to parental groups and meet other parents and children*’ [i3].

The first-time mothers stated that physical parental groups cannot be replaced by digital groups, even though they also acknowledged not knowing what they missed out on.

*‘*… *but just like this when you become a bit united with… well*,* through CHS and such… it seems very fun*,* but… well*,* now I haven’t had it*,* and maybe it would be boring as hell*,* but … yes*,* I can think that is a bit… a bit boring*,* because you become a bit lonely when you are pregnant in a pandemic’* (i8).

### Informal support

Informal support means support from other sources than society during the COVID-19 pandemic. It is characterized by the sub-categories *Support from family and friends* and *Support from social media*.

#### Support from family and friends

Support from family and friends refers to descriptions of how first-time mothers primarily turn to their social network for support with everyday things such as obtaining food and guidance on how to buy shoes. The mothers said to avoid social isolation, they created small social networks despite restrictions; but there were also statements by first-time mothers that they did not feel socially isolated despite the lockdown as they did not need a large social network to feel safe. Support from family refers to first-time mothers’ descriptions of being supported by relatives, parents, and siblings as well as by their partners with whom they discussed thoughts about their children. The support from the first-time mothers’ partners was described as the greatest support during the COVID-19 pandemic. The mothers shared the experience of being first-time parents and described their appreciation of the fact that their partners worked from home. One mother said, ‘*It was also a huge help*,* and it was fantastic! It would have been much more difficult if he had had to be at work like he was before the pandemic’*(i9).

Support from friends refers to the first-time mothers receiving support from other parents and friends with whom they shared common experiences, such as breastfeeding and receiving help from CHS. The first-time mothers described feeling supported when talking to other parents about their children’s personality, for example, shyness, and aspects of development such as language and activities related to their children and how much they should play with their children.

‘*We also have friends who have children… Not very many in our age - or*,* in our child’s age*,* though - I still talked to them on the phone and we saw each other outside sometimes*,* although it wasn’t always easy when it was cold in the winter. We talked a bit*,* but I also noticed then that it is different depending on which phase the child is in*’ [i4].

#### Support from social media

*Support from social media* refers to first-time mothers’ descriptions of their utilisation of digital resources and digital networks with other parents. Digital resources refer to the parents’ descriptions of how they searched for and received answers to questions about parenting, children’s food, sleeping habits, and validation about breastfeeding from the internet. One mother expressed: ‘*the best breastfeeding blog I looked at*,* I actually got tips about from the midwife* [i1]. They described how digital resources had become equivalent to a vast mothers’ group. Nevertheless, they experienced disadvantages of seeking support digitally: they experienced anxiety when information did not match their child.

Digital networks with other parents refer to descriptions of using internet forums to ask questions from other parents in the same situation. First-time mothers said that social media filled a void during the pandemic. For instance, one mother said:

*‘It is probably the internet that I have been supported by a lot*,* but it has also created unnecessary anxiety at the same time for certain things!’* (i3) … *‘you see a lot on social media as well as with those on parental leave who go for pram walks*,* go for coffee together and I think they meet other parents more than I do’ (i3).*

## Discussions

This study revealed that first-time mothers experienced *a gap between their needs for parental support and the support provided*. This gap is described using the categories *Formal support*, *Lack of formal support*, and *Informal support*.

First-time mothers described their need for person-centred support. Such support was received when, for instance, the CH nurses made exceptions to the COVID-19 restrictions by allowing both parents to come to visits at CHS. Similar findings were made by McLeish et al. [24], who further described how mothers felt safe and truly cared for when health care professionals overrode the restrictions in order to adapt the support to the mother’s needs. Further, adapting support to each parent may be referred to as targeted parental support to vulnerable parents [9]. Offering targeted or adapted support to first-time parents own needs may be particularly important during a pandemic as restrictions may reduce social contact and support from family and friends. Previous research highlights that first-time mothers experienced feelings of calm and security about childbirth and parenting when they experienced social support from social networks [25]. In cases where the nurses did not make exceptions to the restrictions, the first-time mothers said they felt completely alone and without support because their partners were not allowed to attend health visits. Experiences of being abandoned and lacking a supporting partner in the health care context during the pandemic were also reported in previous research [2, 4, 24, 26, 27]. Furthermore, the restrictions have been described as leading to inequality between parents [3]. Fathers commonly ask for additional support and inclusion in antenatal and CHS settings [28–30] and parental support to fathers have been reported to contribute to equality between parents [31]. The first-time mothers’ experiences of being alone and missing their partners at child health visits strengthened the belief that there is a need for including both parents in parental support regardless of pandemic restrictions. The fact that nurses made exceptions on their own initiative to adapt to the individual needs of parents contributes to the fact that the support offered to parents is not equally distributed or equally accessible this despite the nurses’ good intention to provide person-centred support. This is also reflecting the findings as opposite experiences.

Furthermore, first-time mothers specifically expressed a desire for person-centred support regarding their mental health needs, which is a part of CH nurses’ assignment [6] and is probably particularly important in uncertain times such as during the COVID-19 pandemic. In line with other research [27], the first-time mothers in this study reported that they were not validated in their desire for mental support. Therefore, the need for support did not correspond to the formal support given. Also, in a non-pandemic context first-time parents ask for individualized and emotional support from child health nurses to facilitate their transition to parenthood [12, 13, 17, 30]. This indicates that the emotional support for first-time parents needs to be strengthened. However, there were also first-time mothers who perceived that CH nurses assumed that the restrictions caused social isolation although that was not the case because the mothers had partners working from home, which made a difference. A conclusion was made that there is room for improvement to develop person-centred support. Further, mothers reported that support from CH nurses was too general and primarily focused on the child, while totally neglecting the parents’ needs. This report was confirmed in previous research [17, 32]. Lefévre et al. [33] found that there is a difference between the expectations of support that parents have and what is provided. This finding strengthened the feeling that parental support needs to be based on parents’ needs, as emphasized in person-centred care [34] and is a prerequisite for the parents to receive the support they request and are entitled to.

Another way to adjust the support provided during the pandemic was through the organization of pram walks by open preschools to facilitate connections among parents. Usually, parental groups in the CHS contribute to such social networks for new parents [35]. There were also further adjustments to provide information and support. However, the first-time mothers in this study reported that adjustments through measures such as information films and digital parental groups did not fully compensate for the absence of physical meetings. Parental groups, besides providing information, offer an opportunity for social interaction [10, 11]. This finding is in line with previous research highlighting that virtual healthcare can make new parents feel concerned rather than safe [36]. During the pandemic with its restrictions on social distancing, digital solutions seemed to be the best alternative due to the specific situation.

During the pandemic, first-time mothers experienced an inconsistent flow of information, and because there were no parental groups to fill the gap due to this inconsistency, the mothers had a sense of insecurity. Adesanya et al. [36] reported that at the beginning of the pandemic, there was a lack of knowledge about how COVID-19 affected pregnancy and breastfeeding, which limited healthcare providers’ ability to provide consistent evidence-based information. Other research describes increased challenges in delivering CH care during the pandemic [5, 18, 19]. This inconsistency may be a natural effect of a pandemic but because the first-time mothers did not have access to parental groups to discuss their uncertainty, their uncertainties may have added further strains on them.

Part of the formal support was reduced or absent during the pandemic [1, 5, 18]. Therefore, first-time mothers reported that they found support in other ways. As stated, the greatest source of support was from their partners. Their partners had the opportunity to work from home due to the COVID-19 restrictions [37]. The first-time mothers said that it was a great support to them for their partners to work from home and share time with their first child. Previous research [37, 38] reported that a partner working from home means additional support. Besides, fathers expressed gratitude for the opportunity of sharing precious moments with their children [2]. Important to note is that the option to work from home does not apply to everyone. As the result shows that the partner’s support is valued so highly, there are social consequences such as parental leave for the partner. Further, this may put vulnerable parents, such as those who are single parents or those who live in dysfunctional relationships, in a more vulnerable position in comparison with those who do not.

Furthermore, first-time mothers obtained informal support by creating a small social network to avoid social isolation despite the COVID-19 restrictions. Previous research confirmed the need for social support and social networks for first-time parents [5, 25, 27, 30, 35]. In line with other research [37] the first-time mothers in this study reported that digital networks filled a void during the pandemic. However, seeking support digitally simultaneously created anxiety and worries when the information did not match their child’s situation, as observed in other research [39]. Therefore, person-centred support is particularly important because it provides evidence-based knowledge that enables an evaluation of the general information online.

### Methodological considerations

This study contributes to research on first-time mothers’ experiences of parental support from CHS in Sweden during the COVID-19 pandemic. The chosen method, an inductive qualitative approach, was suitable for exploring parents’ experiences of parental support during the COVID-19 pandemic and due to the small sample size. The results are presented in generic categories and subcategories with concepts derived from the data, in line with the principles of inductive content analysis [21]. Clear descriptions of the study context, characteristics of participants, data collection and analysis strengthen transferability. To meet the criteria for trustworthiness, the analysis process is described, and citations are used to enable the reader to follow the process [21, 40]. A potential limitation is that this study was conducted in two regions in Sweden and, therefore, the context of the results must be understood. A major limitation is the sample. It was only nine parents participating in the study, all mothers – nor fathers or non-birthing parents. Additionally, most participating mothers were well-educated, and all of them lived with the other parent, potentially influencing their experiences of parental support from CHS. This homogenous group limits the transferability to groups in need of more support, as for example single parents and parents with low education. The small and homogenous sample was due to the difficulties to recruit parents as it was not possible to get support from the CH nurses whose competence and skills were needed to help reduce the spread of COVID-19 in the society by working in vaccination centres. A final but important limitation of this study is that it is difficult to know if all experiences were directly related to the COVID-19 pandemic, as first-time mothers lacked a baseline for comparison.

## Conclusions and clinical implications

This study contributes to an understanding of first-time mothers’ experiences of parental support during the COVID-19 pandemic. The results indicate that during restrictions, the need for person-centred support for both parents is particularly important to ensure that all parents get the support they expect and need. Further, the mothers expressed a desire for adapted parental groups that could operate in spite of the restrictions imposed by the pandemic to be able to connect with other parents and build networks. Research is needed to investigate the future support needs for individuals becoming parents for the first time during the pandemic and explore if their experiences of parental support will affect future family formation.

## Data Availability

The datasets used and analysed during the current study are not publicly available because they contain personally identifiable information; data are available from the corresponding author on reasonable request.

## Abbreviations

CH: Child health
CHN: Child health nurses
CHS: Child health services
